# Acquisition of chopstick-operation skills with the non-dominant hand and concomitant changes in brain activity

**DOI:** 10.1038/s41598-019-56956-0

**Published:** 2019-12-31

**Authors:** Daisuke Sawamura, Satoshi Sakuraba, Yumi Suzuki, Masako Asano, Susumu Yoshida, Toshihiro Honke, Megumi Kimura, Yoshiaki Iwase, Yoshitaka Horimoto, Kazuki Yoshida, Shinya Sakai

**Affiliations:** 1 Department of Functioning and Disability, Faculty of Health Sciences, Hokkaido University, Hokkaido, Japan; 2 Department of Rehabilitation Sciences, Health Sciences University of Hokkaido, Hokkaido, Japan; 3 Department of Occupational therapy, Yamagata Prefectural University of Health Sciences, Yamagata, Japan; 4 Department of Physical Therapy, Chiba prefectural university of health sciences, Chiba, Japan

**Keywords:** Brain imaging, Neurological disorders

## Abstract

Despite their common use as eating utensils in East Asia, chopsticks require complex fine motor-skills for adequate operation and are thus most frequently used with the dominant hand; however, the effect of training time on the proficiency of using chopsticks with the non-dominant hand, as well as the brain activity underlying changes in skill, remain unclear. This study characterised the effect of time spent training in chopstick operation with the non-dominant hand on chopstick-use proficiency and the related brain activity to obtain data that may help individuals who are obliged to change handedness due to neurological disease to learn to use their non-dominant hand in performing daily activities. Thirty-two healthy right-handed students were randomly allocated to training (n = 16) or control (n = 16) groups; the former received 6 weeks of training in chopstick use with their non-dominant (left) hand, and the latter received none. After training, significant improvements in the execution speed and smoothness of upper extremity joints were observed in the training group. Moreover, left dorsolateral prefrontal cortex activity significantly decreased, and bilateral premotor cortex activity significantly increased across training. These results indicated that 6 weeks of chopstick training with the non-dominant hand effectively improved chopstick operation.

## Introduction

Approximately 70–80% of stroke survivors suffer from upper extremity paralysis, which compromises the motor skills necessary for activities of daily living (ADL)^1,2^. Moreover, as about 80% of humans are right-handed and 45–50% of strokes occur in the left hemisphere^3,4^, many stroke survivors are obliged to change handedness; this represents a compensatory strategy to execute daily skills specific to the dominant hand (e.g., writing and eating meals). To achieve this goal, patients must perform repetitive motor-learning exercises with their non-dominant hand. In some Asian countries, eating with chopsticks is an important ADL that requires fine motor-skills, and intensive, repetitive training may be required to acquire this skill with the non-dominant hand. However, the mechanism and neural activity underlying the learning of handling chopsticks with the non-dominant hand and the effect of training time on the acquisition of this skill are not yet completely understood.

One meta-analysis has demonstrated the effect of motor-learning on neural activities related to motor-skill acquisition^5–9^. Motor-skills are commonly assessed in terms of movement speed, limb geometry, accuracy, and movement consistency^6,8^; changes in these parameters are classified as either acquisition or retention, depending on their duration.

The motor-learning theory of Fitts and Posner (1967)^10^ emphasises the importance of long-lasting changes in acquired skills. Specifically, the theory segments motor-learning into three sequential stages—cognitive, associative, and autonomous—that demand increasingly less conscious attention on task performance as motor-skills improve.

Multiple neuroimaging studies have provided evidence supporting this motor-learning model^11–14^. Functional magnetic resonance imaging (fMRI) and positron-emission tomography/computed tomography studies have reported that the dorsolateral prefrontal cortex (DLPFC), primary motor cortex (M1), and supplementary motor area (SMA) play important roles in the early phase of motor-learning, which gradually diminish across the motor-learning process^11,12^. On the other hand, activity in the premotor cortex (PMC), SMA, parietal regions, striatum, and cerebellum gradually increase as motor-skills improve^11,13,14^. These results suggest that regional activities shifted from cortical areas related to intentional or endogenous attention to those associated with motor programming and the regulation of motor output^15^.

Increasingly more research on motor-learning has used neuromodulatory, non-invasive brain stimulation, such as transcranial direct current stimulation (tDCS), to modify cortical excitability. Indeed, Buch *et al*.^16^ reported that the number of tDCS studies rose by more than 60% from 2013 to 2015. tDCS stimulation of the primary motor cortex^17–19^, PMC (especially the dorsal PMC)^20–22^, and PFC has indicated the involvement of these areas in motor-learning^19,21^. Several studies of tool-related motor-skill learning, such as writing^23^, golf putting^24^, and ball rotation^25^, provide further evidence for the recruitment of the aforementioned areas in motor-learning.

Functional near-infrared spectroscopy (fNIRS) can obtain measurements of brain activity with high ecological validity, including neural activity during motor-learning^26^. On the other hand, fNIRS studies are frequently limited by small sample sizes, the absence of randomisation, and their use of task paradigms that are reproducible with fMRI^26^. While no fNIRS study has yet explored brain activity associated with the motor-learning of chopstick use with the non-dominant hand, an fMRI study has revealed that the resting-state functional connectivity between the parietal cortex, sensory-motor cortex, and cerebellum significantly decreased after 8 weeks of training in the handling of chopsticks with the non-dominant hand^27^. Task-related brain activity was also measured while subjects repeated simple hand opening and closing movements with chopsticks (skill was not required as the two sticks were connected), but no significant difference in task-induced activation was found between pre- and post*-*training. As the authors of that study admitted, changes in neural activity underlying the improvement of chopstick operation may have been too small to detect, as the task did not require rigorous chopstick-operation skills. Moreover, the simplified task may not reflect brain activity that corresponds to actual chopstick operation, and additionally, the study’s sample size was inadequate.

The present study aimed to elucidate the effect of training time in use of the non-dominant hand on brain activity, by assessing the brain activity during chopstick operation with the non-dominant hand in groups with and without training, across a 6-week training period. Considering the recommendation of a previous study^26^, we employed fNIRS and adopted a single-blinded and randomised study design to ensure ecological validity. Additionally, we focussed on the relationship between task proficiency and the training period, which is highly dependent on the nature of task. Elucidating the effects of, and neural activation resulting from chopstick training with the non-dominant hand under ecologically valid conditions would provide the data necessary to establish an effective rehabilitation approach for patients who must change their handedness on account of stroke or other diseases.

## Results

### Demographic data

There were no differences in demographic data, including age (training and control groups, 21.56 ± 0.81 and 21.50 ± 0.73 years, respectively), sex (8 women in both groups), and the Edinburgh Handedness Questionnaire Inventory score (training and control groups, 90.63 ± 7.72 and 90.63 ± 7.83, respectively) between the control and training groups at baseline.

### Speed of chopstick task

**Figure 1:**
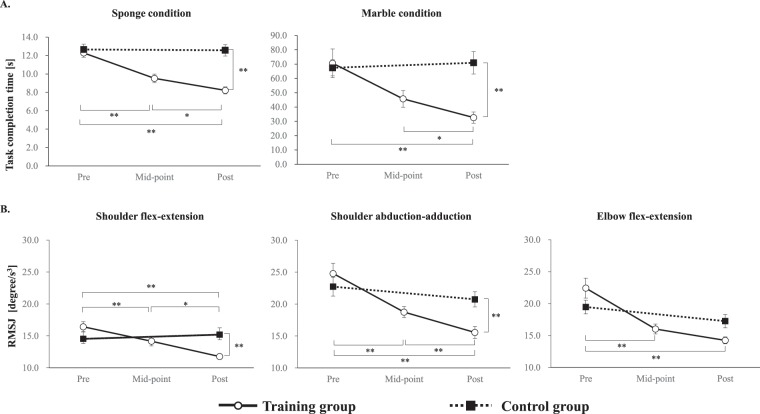
Changes in behavioural data across training. (**A**) The time required to move five objects for sponge transferral (left) and the marble condition (right). (**B**) The root mean square jerk (RMSJ) in shoulder flexion–extension (left), in shoulder abduction–adduction (middle), and in elbow flexion–extension (right). *p < 0.01; **p < 0.001. Error bars indicate the standard error.

A 2 × 2 mixed design analysis of variance (ANOVA) of the time taken to transfer an item (a sponge) with chopsticks used in the non-dominant hand, with time (pre-training vs. post-training) as the within-subjects factor, and group (training group vs. control group) as the between-subjects factor, revealed a significant main effect of group (F(1,30) = 12.89, p < 0.01) and time (F(1,30) = 28.16, p < 0.01) and a significant group × time interaction (F(1,30) = 25.79, p < 0.01). A post-hoc *t*-test showed that the task completion time in the training group (mean task completion time, 8.21 ± 1.54 s) was significantly shorter than that in the control group (mean task completion time, 12.57 ± 2.50 s) at the post-training assessment (t(30) = 5.76, p < 0.01). A one-way ANOVA with time (pre-training, mid-point, and post-training) as a factor revealed a significant main effect (F(2,30) = 31.21, p < 0.01) in the training group. A post hoc test with Bonferroni correction revealed significant differences between all time-points (pre-training vs. mid-point: t(15) = 4.72, p < 0.01; pre-training vs. post-training: t(15) = 8.00, p < 0.01; mid-point vs. post-training: t(15) = 2.73, p = 0.047; Fig. 1A and Supplementary Table 1).

In a 2 × 2 mixed design ANOVA of the time required to transfer a marble using chopsticks in the non-dominant hand, we found significant main effects of group (F(1,30) = 5.88, p = 0.02) and time (F(1,30) = 5.70, p = 0.02), and a significant group × time interaction (F(1,30) = 8.27, p < 0.01). A post hoc *t*-test showed that the task completion time in the training group (mean task completion time; 32.60 ± 15.76 s) was significantly shorter than that in the control group (mean task completion time; 70.96 ± 31.39 s) at the post-training assessment (t(30) = 4.37, p < 0.01). One-way ANOVA with time (pre-training, mid-point, and post-training) as a factor revealed a significant main effect (F(2,30) = 10.48, p < 0.01) in the training group, and a post hoc test with Bonferroni correction revealed significant differences between post-training and pre-training/mid-point (pre-training vs. post-training: t(15) = 3.81, p < 0.01; mid-point vs. post-training: t(15) = 2.78, p = 0.04; Fig. 1A and Supplementary Table 1).

### Smoothness of joint movement during chopstick operation

To assess smoothness of joint movement during chopstick operation in the non-dominant hand, we performed 2 × 2 mixed-design ANOVA of the root mean square of angular jerk (RMSJ) of left-shoulder flexion–extension, with time (pre-training vs. post-training) as a within-subjects factor and group (training group vs. control group) as a between-subjects factor. We found a significant main effect of time (F(1,30) = 9.045, p < 0.01) and a significant group × time interaction (F(1,30) = 5.833, p < 0.01). A post hoc *t*-test showed that the RMSJ of left-shoulder flexion–extension in the training group (mean RMSJ: 11.77 ± 1.32 degree/s^3^) was significantly lower than that in the control group (mean RMSJ: 15.20 ± 3.20 degree/s^3^) at post-training assessment (t(30) = 4.20, p < 0.01). One-way ANOVA with time (pre-training, mid-point, and post-training) as a factor revealed a significant main effect (F(2,30) = 18.61, p < 0.01) in the training group, and a post hoc test with Bonferroni correction revealed significant differences between all time-points (pre-training vs. mid-point: t(15) = 2.70, p = 0.049; pre-training vs. post-training: t(15) = 5.56, p < 0.01; mid-point vs. post-training: t(15) = 4.07, p < 0.01; Fig. 1B and Supplementary Table 1**)**.

In a 2 × 2 mixed-design ANOVA of the RMSJ of left-shoulder abduction–adduction and left-elbow flexion–extension, we observed significant main effects of time (shoulder abduction–adduction: F(1,30) = 22.92, p < 0.01; elbow flexion–extension: F(1,30) = 21.60, p < 0.01) and a significant group × time interaction (shoulder abduction–adduction: F(1,30) = 10.47, p < 0.01; elbow flexion–extension: F(1,30) = 5.83, p = 0.02). Post hoc tests showed that the RMSJ of left-shoulder abduction–adduction in the training group (mean RMSJ: 15.56 ± 3.75 degree/s^3^) was significantly lower than that in the control group (mean RMSJ: 20.75 ± 4.77 degree/s^3^) at the post-training assessment (t(30) = 3.63, p < 0.01), but there was no significant difference in the RMSJ of left-elbow flexion–extension at any time-point. One-way ANOVA with time (pre-training, mid-point, and post-training) as a factor revealed a significant main effect of time in shoulder abduction–adduction (F(2,30) = 21.00, p < 0.01) and in elbow flexion–extension (F(2,30) = 19.27, p < 0.01). A post hoc test with Bonferroni correction revealed significant differences in the training group between all time-points (all, p < 0.01) in shoulder abduction–adduction and between pre-training and mid-point/post-training in elbow flexion–extension (all, p < 0.01; Fig. 1B and Supplementary Table 1).

### Brain activity during chopstick operation

**Figure 2:**
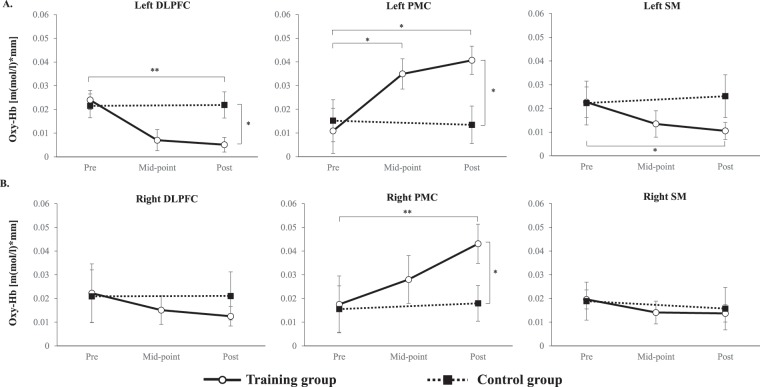
Changes in Oxy-Hb concentration in each ROI across training. (**A**) Changes in Oxy-Hb concentration in the left DLPFC (left), left PMC (middle), and left SM (right). (**B**) Change in Oxy-Hb concentration in the right DLPFC (left), right PMC (middle), and right SM (right). Oxy-Hb: oxygenated haemoglobin; ROI: region of interest; DLPFC: dorsolateral prefrontal cortex; PMC: pre-motor cortex; SM: primary sensory motor cortex. *p < 0.01; **p < 0.001. Error bars indicate the standard error.

The channels in which significant activation was detected (p < 0.05, FDR corrected) during the chopstick-operation task were compared with their baseline activity (Supplementary Fig. 1). A 2 × 2 mixed-design ANOVA of the left DLPFC, with time (pre-training vs. post-training) as a within-subjects factor and group (training group vs. control group) as a between-subjects factor, revealed a main effect of time (F(1,30) = 4.28, p = 0.047) and a significant group × time interaction (F(1,30) = 4.19, p = 0.049). A post hoc *t*-test revealed significantly low oxygenated haemoglobin (Oxy-Hb) concentration in the left DLPFC of the training group relative to that of the control group (t(30) = 2.73, p = 0.01) at the post-training assessment. One-way ANOVA with time (pre-training, mid-point, and post-training) as a factor revealed a significant main effect of time (F (2,30) = 6.81, p < 0.01) in the training group. A post hoc test with Bonferroni correction revealed a significant difference in DLPFC activity between pre-training and post-training in the training group (t(15) = 4.06, p < 0.01), but not in the control group. (Fig. 2A and Supplementary Table 1).

In terms of Oxy-Hb concentrations in the left dorsolateral premotor cortex (dPMC), a 2 × 2 mixed design ANOVA revealed a significant group × time interaction (F(1,30) = 4.26, p = 0.048), and a post hoc *t*-test revealed significantly higher Oxy-Hb concentration in the training than in the control group (t(30) = 2.74, p = 0.01) at the post-training assessment. One-way ANOVA with time (pre-training, mid-point, and post-training) as a factor revealed a significant main effect of time (F(2,30) = 8.94, p < 0.01) in the training group. The post hoc test with Bonferroni correction revealed that the Oxy-Hb concentration at post-training was significantly higher than at pre-training (t(15) = 3.44, p = 0.01) and that the Oxy-Hb concentration at mid-point was significantly higher than at pre-training in the training group (t(15) = 3.04, p = 0.03; Fig. 2A and Supplementary Table 1).

Regarding neural activity in the right dPMC, a 2 × 2 mixed-design ANOVA revealed a significant main effect of time (F(1,30) = 4.58, p = 0.041) and a significant group × time interaction (F(1,30) = 4.43, p = 0.044). A post hoc *t*-test revealed significantly higher Oxy-Hb concentration in the training than in the control group (t(30) = 2.92, p < 0.01) at the post-training assessment. One-way ANOVA with time (pre-training, mid-point, and post-training) as a factor revealed a significant main effect of time (F(2,30) = 5.78, p = 0.02) in the training group. A post hoc test with Bonferroni correction revealed that the Oxy-Hb concentration at post-training was significantly higher than at pre-training (t(15) = 3.37, p = 0.01; Fig. 2B and Supplementary Table 1).

**Figure 3:**
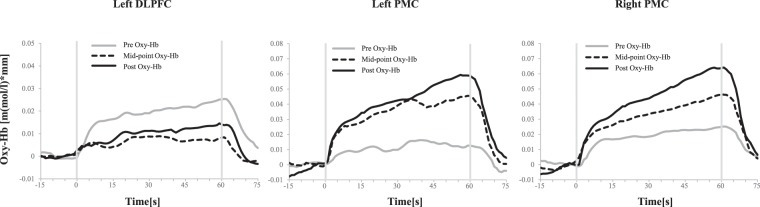
Time course of changes in average Oxy-Hb concentration during a motor execution task in the left DLPFC (left), left PMC (middle) and right PMC (right). The horizontal axis represents time, and the grey vertical line represents task onset (0 s) and end (60 s). Oxy-Hb: oxygenated haemoglobin; DLPFC: dorsolateral prefrontal cortex; PMC: pre-motor cortex; SM: primary sensory motor cortex.

In the right DLPFC and bilateral primary sensory motor cortex (SM), no significant main effect or interaction was found. One-way ANOVA revealed no significant main effect of time in the training group. The time course of mean Oxy-Hb concentration in the training group in the left DLPFC, left dPMC, and right dPMC are shown in Fig. 3; these areas featured a significant group × time interaction as revealed by the 2 × 2 mixed design ANOVA.

### Correlation analysis

**Figure 4:**
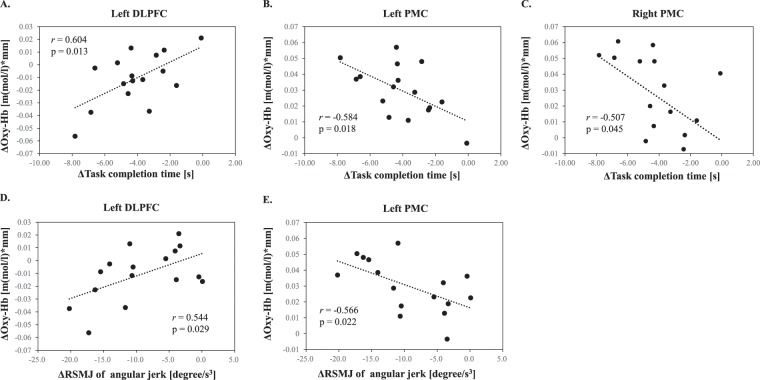
Correlation analysis between ΔOxy-Hb concentration in each ROI and Δbehavioural data. (**A**) A significant positive correlation was found between ΔOxy-Hb concentration in left DLPFC and Δtask completion time for sponge transferral. (**B**) A significant negative correlation was found between ΔOxy-Hb concentration in left PMC and Δtask completion time for sponge transferral. (**C**) A significant negative correlation was found between ΔOxy-Hb concentration in right PMC and Δtask completion time for sponge transferral. (**D**) A significant positive correlation was found between ΔOxy-Hb concentration in left DLPFC and ΔRMSJ of shoulder abduction–adduction. (**E**) A significant negative correlation was found between ΔOxy-Hb concentration in left PMC and ΔRMSJ of shoulder abduction–adduction. Oxy-Hb: oxygenated haemoglobin; ROI: region of interest; Δ: change; DLPFC: dorsolateral prefrontal cortex; PMC: pre-motor cortex; SM: primary sensory motor cortex; RMSJ: root mean square angler jerk.

We found a significant positive correlation between ΔOxy-Hb in the left DLPFC and Δtask completion time for sponge transferral, and between ΔOxy-Hb in the left DLPFC and ΔRMSJ in shoulder abduction–adduction (ROI; Fig. 4). On the other hand, the ΔOxy-Hb in the left dPMC was significantly negatively correlated with Δtask completion time for sponge transferral and with the ΔRMSJ in shoulder abduction–adduction. Moreover, we observed a reliable trend (r = −0.50; p = 0.05) in the Δtask completion time for marble transferral. Additionally, the ΔOxy-Hb in the right dPMC was significantly negatively correlated with Δtask completion time for sponge transferral.

Furthermore, we performed a correlation analysis between the two chopstick-operation skills (speed and smoothness), pre- and post-assessment, in the training group (Supplementary Table 2). We found a significant positive correlation between task completion time for sponge transferral and the RMSJ in shoulder abduction–adduction at pre-assessment (r = 0.54; p = 0.03). We also found significant positive correlations between task completion time for sponge transferral and RMSJ in shoulder flexion–extension (r = 0.54; p = 0.03) and abduction–adduction (r = 0.74; p < 0.01) at post-assessment. Similarly, we found significant positive correlations between task completion time for marble transferral and the RMSJ in elbow flexion–extension (r = 0.55; p = 0.03) and shoulder abduction–adduction (r = 0.54; p = 0.03) at post-assessment (Supplementary Table 2).

## Discussion

### Chopstick operation skill

The training group completed both the marble and sponge transferral tasks in a significantly shorter time and had a significantly lower RMSJ in shoulder flexion–extension and shoulder abduction–adduction at the post-training assessments than at the mid-point or pre-training assessments. These results suggest that the 6-week chopstick operation training with the non-dominant hand improved the speed and smoothness of chopstick operation with the non-dominant hand. Moreover, the task completion time for sponge transferral in the training group significantly decreased as training progressed; however, this effect was not observed for marble transferral between the pre-training and mid-point assessments. These results may reflect a difference in task difficulty between moving the two types of objects; in a previous review of the effects of various motor-learning tasks and associated neural plasticity, Dayan and Cohen^15^ reported that the efficiency of motor-skill learning is task-dependent and that more time is required to acquire more complex or finer motor-skills. Additionally, the large variability in task completion time among the participants indicated that the reliability of the marble condition as a measure of chopstick-operation skill was less than that of the sponge condition. This low reliability may have affected the task completion time.

Regarding the smoothness of upper extremity joint movement, the RMSJ in shoulder flexion–extension and abduction–adduction decreased significantly as training proceeded; however, there was no significant decrease in the RMSJ in elbow flexion–extension between the mid-point and post-training assessments in the training group.

The relationship between task difficulty and the time needed to acquire a skill may account for these results. Dayan and Cohen (2011)^15^ modelled the acquisition of skill proficiency as a progression from fast to slow motor-skill learning. The elbow joint is a hinge joint with a uniaxial direction of motion, whereas the shoulder joint is a spherical joint capable of multi-axis motion. Early improvements in the speed and smoothness of elbow-joint motion indicates that participants could control the elbow joint with greater ease; hence, the learning stage of an elbow-joint motion might have already shifted to the slow motor-skill learning stage by the mid-point and post-training assessments.

### Brain activity

While the activation of the left DLPFC in the training group was significantly lower at the post-training than at the pre-training assessment, the Oxy-Hb concentration in the bilateral dPMC of the training group was significantly higher at the post-training than at the pre-training assessment. Previous studies have revealed that the DLPFC plays an important role in top-down attention control and in executive functions, such as planning and problem-solving^28–34^. In motor-learning, DLPFC activity is related to cognitive control of sensory input and future action planning in the fast motor-learning stage^35,36^, as well as in the encoding of declarative memory^37,38^. A meta-analysis of neuroimaging studies on motor-learning divided motor training periods into three phases, including short-term (≤1 h), medium-term (>1 h and ≤24 h), and long-term (24 h to <5 weeks) periods^6^. This previous study also reported a relationship between training time and brain activity: as training time increased, bilateral DLPFC activity decreased. Kerns *et al*.^39^ reported that the difference between neural activity in the left and right DLPFC during the motor-learning process reflects differences in control processes that depend on the task paradigms. Chopstick operation with the non-dominant hand is a tool-use motor-learning task; previous studies have observed that specific brain activity patterns in the left hemisphere are independent of the handedness of tool-use^40–42^, and the demonstration of the DLPFC as an important region in tool-use behaviour suggests that the increased activity observed in the left DLPFC during the early phases of chopstick-operation training is related to intentional tool use^41,43^. Furthermore, decreases in left DLPFC activation might more sensitively reflect the improvement in motor-skills than changes in right DLPFC activation.

Bilateral dPMC activity in the training group significantly increased from the pre-training to the post-training assessments. The dPMC plays an essential role in motor control and learning of goal-oriented action and receives integrated visual and somatosensory information from medial intraparietal areas that is used to plan arm movement trajectories^44^. Multiple studies have demonstrated the important role of the dPMC in motor-learning^20–22,45^. Our findings concerning bilateral dPMC activity are consistent with these neuroimaging studies. Furthermore, a meta-analysis of motor-learning studies has demonstrated that dPMC activity features hemispheric lateralisation that is not solely attributable to differences in handedness^8^. This meta-analysis study suggested that the left dPMC plays a particularly important role in motor sequence and sensory motor-learning. Having considered inter-hemispheric specificity, the study suggested that left dPMC activity is associated with unilateral motor performance of either hand and that the right dPMC is associated with the actions of both hands^46^. Previous studies have also reported that, while the left dPMC plays a prominent role in the integration of visual, sensory, and motor information, the right dPMC may only provide support^8,47–49^. Use of the non-dominant hand to operate chopsticks requires visual, sensory, and motor integration. Therefore, the left dPMC might be involved in in the use of chopsticks with the left hand.

While activity in the left dPMC significantly increased at the mid-point assessment, right-dPMC activation only increased slightly over the same period. The left dPMC is reportedly involved in sequence acquisition; and the right dPMC, in storage of sequences and advanced learning^50^. The increased activity in the right dPMC at post-training assessment could indicate that chopsticks operation with the non-dominant hand had achieved an advanced stage of proficiency. Although our chopstick operation task differed from the nature of the tasks employed by Schubotz *et al*.^50^, the difference in activation times between the left and right dPMCs might provide further support for their findings. Another possibility is that the significant increase in left dPMC activity at the mid-point assessment reflects tool-use behaviour, the same explanation proposed to account for the changes in left DLPFC activity.

### Correlations between chopstick-operation skill and brain activity

We observed a significant positive correlation between ΔOxy-Hb concentration in the left DLPFC and Δtask completion time for sponge transferral and between ΔOxy-Hb concentration in the left DLPFC and ΔRMSJ in shoulder abduction–adduction. Significant negative correlations were observed between ΔOxy-Hb concentration in the bilateral dPMC and Δtask completion time for sponge transferral, and between the ΔOxy-Hb concentration in the left dPMC and ΔRMSJ in shoulder abduction–adduction. Additionally, the ΔOxy-Hb concentration in the left dPMC had a reliable negative trend (r = −0.50; p = 0.05) with respect to Δtask completion time for marble transferral. These findings suggest that task-completion time during sponge transferral, which lacked a direct relationship with brain activity during sponge transferral, correlated closely with the changes in brain activity during marble transferral task; while we observed a direct association between task-completion time and brain activity during marble transferral, this relationship was weaker than the aforementioned association across objects. The large variability in task completion time for the marble condition may have had a major impact on these results. These significant cross-object correlations may suggest that the marble transferral task broadly reflects not only object-specific skill, but also the more general chopstick-operation skill with less dependence on task difficulty. These results concerning the relationships between chopstick-operation skills and brain activity are consistent with a previous review of changes in behaviour and brain activity during fast motor-skill learning^15^, which reported that bilateral DLPFC activity decreased and bilateral PMC activity increased alongside rapid and remarkable skill improvement during fast motor-skill learning. Consequently, motor-skill learning level and brain activity observed at the post-training assessment may correspond to the fast motor-skill learning stage rather than to a progression to the slow motor-skill learning stage.

Additionally, Kantak *et al*.^44^ reported that the dPMC is critical for associative learning, where an internal representation is developed, with practice, between arbitrary yet behaviourally relevant cues and appropriate motor commands. Therefore, the activity in the left DLPFC and bilateral dPMC may indicate the degree of motor-skill learning with the non-dominant hand during the fast motor-skill learning period. Additionally, we found significant positive correlations between the two chopstick-operation skills (speed and smoothness) in the training group at post-assessment. These results may indicate the close relationship between chopstick-operation and smoothness of the upper extremity joint movement in response to the progress of motor-skill learning and may support the interpretation of changes in brain activity as being consequent of motor-skill learning.

### Conclusions and future directions

This single-blinded, randomised study explored the effects of training in the use of chopsticks – a practical skill that is often used in daily life – with the non-dominant hand on the speed and smoothness with which chopsticks are used, as well as on brain activity underlying the improvement of proficiency in chopstick operation. Chopstick operation requires complex and fine hand and upper extremity motor skills, which are gradually honed by repeated practice over many years. Although rigor is required to relearn chopstick use with the non-dominant hand, this study revealed the effect of training time on improving chopstick use with the non-dominant hand and documented concomitant changes in brain activity; this had not been reported previously. Specifically, left-handed chopstick-operation skill and brain activity at the post-training assessment suggested that the acquired proficiency remained in the fast motor-skill learning stage, further indicating that chopstick operation skill with the non-dominant hand can improve with additional training. These data may be of use in helping patients who are obliged to exchange handedness to perform complex motor tasks with their non-dominant hands due to cerebral vascular accidents or other events.

This study was subject to several limitations. Firstly, because fNIRS has a low spatial resolution and is mainly restricted to the measurement of cerebral haemodynamics, we could not evaluate changes in the activity of deep brain regions that contribute significantly to motor-learning, such as the basal ganglia or the cerebellum. Moreover, we could not measure the temporal, occipital, or posterior parts of the parietal region because our method was restricted to 52 channels. However, we were able to use fNIRS to measure DLPFC and PMC activity, which have previously been associated with motor-learning. Secondly, we had not considered that the performance of the experimental task—the time taken to move a specific number of items—during the performance of fNIRS would vary so markedly among participants. Thirdly, we evaluated the smoothness of upper extremity joint movement and measurement of brain activity under the one condition only (marble transferral). Therefore, we cannot generalise these results to other object conditions (e.g., it is possible that Oxy-Hb concentration during sponge transferral could be lower than that for marble transferral). However, we found a significant cross-object correlation between changes in the task performance for sponge transferral and the brain activity measured during marble transferral. Fourthly, the participants in this study had all used chopsticks for many years with their dominant (right) hand; thus, the action model was already stored as memory, and we were unable to consider how this experience might have affected the acquisition of chopstick-operation skills with the non-dominant left hand. Fifthly, study focused on how chopstick training with the non-dominant hand affects motor-skills and brain activity and how this effect varied across training. Therefore, we cannot comment on the application of the skill-specific effects of chopstick operation training to non-chopstick-related training.

Future research should perform longer-term examinations and compare the training protocol results with an active control. Future work should also examine how the effect of training changes over time with age and across diseases that require affected individuals to change their handedness, including stroke, traumatic brain injury, upper limb trauma, amputation, and peripheral nerve disorder. In addition, future studies should investigate how extrinsic feedback provided through occupational therapy or non-invasive brain stimulation might promote the acquisition of chopstick-operation skills with the non-dominant hand as compared to repetitive implicit training alone.

## Methods

### Participants

Thirty-two healthy right-handed participants were recruited in this study (16 women; mean age, 21.7 ± 1.3 years). All participants achieved a score >70 points on the Edinburgh Handedness Questionnaire Inventory^51^ and had no history of neurological or psychiatric disorders. The study protocol was approved by the Ethics Committee of the Health Sciences University of Hokkaido (Approval number:16R033032), and all experiments were performed in accordance with the latest version of the Declaration of Helsinki. All participants provided written informed consent before the experiment.

### Experimental procedure

**Figure 5:**
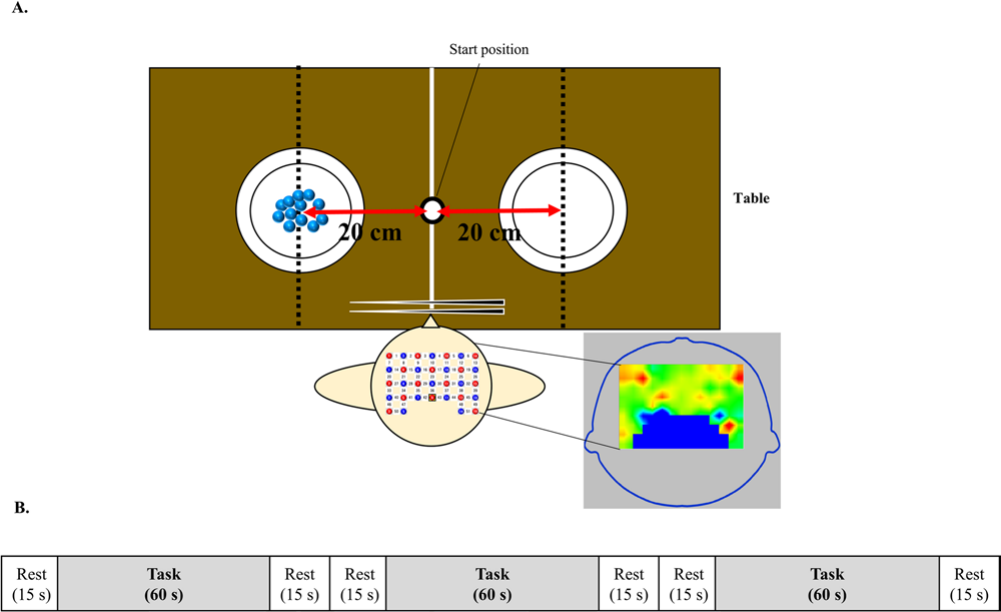
Experimental settings. (**A**) Experimental setting of the chopstick task. Two plates were placed on the table at 20 cm to the left and right of the participants’ midline. (**B**) The experimental design of functional near-infrared spectroscopy measurements. Participants repeated the 60-s marble transferral task thrice. Participants rested for 15 s before and after each task.

This was a single-blinded and randomised study with stratification. After giving consent, participants were stratified according to age, sex, and their Edinburgh Handedness Questionnaire Inventory scores. Stratified randomization was used to allocate each participant to either the training group (n = 16; 8 women; mean age, 21.1 ± 0.7 years) or the control group (n = 16; 8 women; mean age, 21.3 ± 0.8 years) by a researcher who was neither an evaluator nor a trainer. The training group received 6 weeks of training in the left-handed operation of chopsticks at our laboratory for 30 minutes a day, 5 days a week. The control group received no training. The training group practiced chopstick operation with their left hand by repeatedly transferring various objects (e.g., beans, marbles, and sponges) differing in size, weight, shape, and materials from one dish to another. Evaluations of chopstick-operation skill and brain activity were conducted before training (pre-training), after 3 weeks of training (mid-point), and after 6 weeks of training (post- training); the control group only underwent evaluations before (pre-training) and after the 6-week period (post-training) (Supplementary Fig. 2**)**. While evaluations for each participant were conducted separately, they were all performed in the same environment and on the same day. The evaluation environment is presented in Fig. 5A. The mid-point and post-training assessments were conducted at 24–72 h after training to evaluate the retention of chopstick-operation skills. Chopstick-operation skills were evaluated in terms of speed of object transfer and smoothness of upper extremity movement. After evaluation of chopstick-operation skills, there was a 5-minute break followed by measurement of brain activity during the chopstick-operation task using fNIRS. The smoothness of upper extremity movement and measurement of brain activity were evaluated during the marble transferring task.

### Chopstick operation task

#### Speed of motor-execution

The time taken to transfer an object from one dish to another with 21-cm wooden chopsticks was measured as an indication of execution speed. Two round dishes (diameter, 13 cm; height, 5) were placed 20 cm to the left and right of the midline of the participants, who sat on a chair and held the chopsticks in a vertical orientation 15 cm ahead of their body midlines, as the starting position. The participants then started to transfer the objects from the left dish to the right dish using the chopsticks in their left hands. The time taken to transfer five objects from the left dish to the right dish was measured. To ensure the reliability of the measurement, this task was repeated three times with sponges and marbles, and the execution time was averaged across trials for any given object. To evaluate the task performance while considering the difficulty level, we conducted both the marble task (high difficulty) and the sponge task (low difficulty).

#### Quality of operation

We measured the smoothness of upper extremity joint movement during chopstick use as an indication of the quality of operation. A three-dimensional motion analysis device (myoMOTION™, Noraxon, Scottsdale, AZ, USA) was used to quantify angular changes of selected joints by placing motion sensors on body segments. In this study, three motion sensors were placed on three body segments for each individual: the upper thoracic (below C7 in line with the spinal column), upper arm, and forearm. A rigid link model of the three segments was created by three inertia sensors, and the joint angle changes were calculated from the amount of rotation of the inertial sensor attached to each segment in reference to the earth frame. The calculation of each joint angle conformed to the recommended methodology of the International Society of Biomechanics^52^.

The sampling rate was set to 100 Hz. The target upper extremity joint movements included left-shoulder adduction and abduction, left-shoulder flexion and extension, and left-elbow flexion and extension. This measurement was performed during the marble transferral task and was repeated thrice.

The smoothness of upper extremity joint movement was assessed from the time when an object was picked up from the left dish to when it was placed in the right dish. To represent the smoothness, we calculated angular jerk, which is the time derivative of the angular acceleration of a given joint. The RMSJ was then calculated using the following equation:

$$RMSJ=\sqrt{\frac{1}{N}\mathop{\sum }\limits_{i=1}^{N}\,J{(i)}^{2},}$$

where J(*i*) is the jerk of the *i*-th data point, and N is the total number of data points sampled from a given participant.

### Functional near-infrared spectroscopy instrument

**Figure 6:**
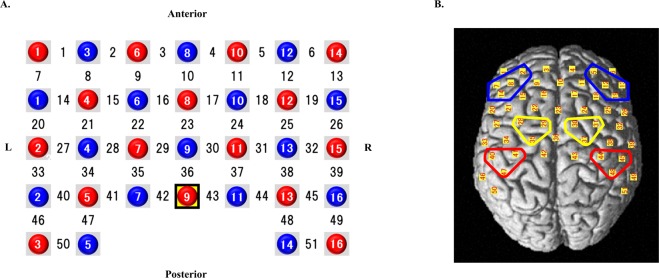
Near infra-red spectroscopy (NIRS) probe arrangement. (**A**) Illuminators are shown as red circles, detectors are shown as blue circles, and channels are shown without any circles. Illuminator probe 9 was placed at Cz according to the international 10/20 placement system. (**B**) The channel positions are shown on the cortical surface. Red, yellow, and blue frames show the sensory-motor area, dorsal premotor area, and dorsolateral prefrontal cortex area, respectively.

The changes in Oxy-Hb concentration was measured with a multi-channel fNIRS optical topography system (LABNIRS, Shimadzu Corp. Kyoto, Japan), using three wavelengths of near-infrared light (780, 805, and 830 nm). The sampling rate was 14.8 Hz. Cz was defined according to the International 10–20 placement system. Probe 9 (between channels 42 and 43) was placed over Cz, and channels 10, 23, and 36 overlapped at the medial line. fNIRS probes consisted of 16 illuminating and 15 detecting probes arranged alternately, with an inter-probe distance of 3 cm, resulting in 51 channels. These probes were placed over the DLPFC, dPMC, and SM areas. The positions of the probes and channels are shown in Fig. 6A. Channels 10, 23, and 36 were excluded from the analysis because these channels were placed on the cerebral longitudinal fissure; thus, only 48 channels were analysed. fNIRS optode positions and reference positions (Cz, Nz, Iz, AL, and AR) were digitized using a three-dimensional digitizer (FASTRAK; Polhemus, Colchester, VT, USA). The coordinate data were registered into Montreal Neurological Institute (MNI) coordinates using the “coordinate-based system” function in NIRS_SPM. The anatomical location of each channel was determined according to the Talairach Daemon^53,54^. Anatomical labelling (Brodmann areas, Talairach Daemon), which was averaged in all participants, is listed for each channel in Supplementary Table 3. Although fNIRS could quantify changes in Oxy-Hb, deoxygenated Hb, and total Hb concentration, all analyses were performed on changes in Oxy-Hb concentration, because this provides the most representative indication of brain activity^55,56^. The baseline period encompassed the 6-s period before task onset, and the average Oxy-Hb value of the baseline period was set as zero. To avoid NIRS pathlength issues, the changes in Oxy-Hb concentration during the task was calculated as the difference from the baseline value^57^. In addition, independent component analysis was applied to remove baseline drift and internal and external noise. A bandpass-filter was then applied between 0.01 and 0.70. The protocol consisted of three blocks, each of which was comprised of the three following phases: (1) 15 s, rest; (2) 60 s, marble transferral task; (3) 15 s, rest (Fig. 5B).

### Statistical analysis

The indices of motion speed and smoothness and the change in Oxy-Hb concentration were analysed using a 2 × 2 mixed-design analysis of variance (ANOVA) with group (training or control) and time (pre-training or post-training) as between- and within-subject factors. To elucidate the relationship between the level of proficiency and the training period, a one-way ANOVA was performed with time (pre-training, mid-point, post-training) as a within-subject factor. Bonferroni correction was applied as a post hoc analysis. To identify the activated channels during the task, the Oxy-Hb concentration data obtained during task performance at pre-training, mid-point, and post-training were compared with those observed at baseline, using paired *t*-tests. The false discovery rate (FDR) method was applied to correct the false positive rate among the channels with significant difference^58^.

Activated channels were grouped into six regions of interest (ROIs) based on the FDR-corrected results from the paired *t*-tests as well as previous study findings^17–22^, and a signal-averaging technique was applied to each ROI to allow further analysis. The six ROIs included the bilateral DLPFC (left: channels 2, 7, 8, and 14 were averaged; right: channels 5, 12, 13, and 19 were averaged), the bilateral dPMC (left: channels 28, 29, and 35 were averaged; right: channels 30, 31, and 37 were averaged), and the bilateral SM (left: channel 40, 41, and 47 were averaged; right: channel 44, 45, and 48 were averaged) (Fig. 6B). Task-related changes in Oxy-Hb concentration in each ROI were calculated by referencing the average of the Oxy-Hb concentration measured in the three blocks to that obtained during the 5-s baseline period (−5 to 0 s). For the subsequent ROI analysis, task-related changes in Oxy-Hb concentration in each ROI were averaged for the time-period during the task (0–60 s after task onset). All channels included in these ROIs were over 60% of the estimated probability in individual-level registration, which indicated the validity of this procedure for ensuring the accuracy of spatial registration. The dPMC regions formed part of Brodmann area 6, and we selected three dorsal channels in each hemisphere as ROIs for the dPMC. The changes in Oxy-Hb concentration in each ROI were analysed using a 2 × 2 mixed-design ANOVA with group and time as factors. In the training group, one-way ANOVA with time (pre-training, mid-point, or post-training) as a factor was performed. In addition, we calculated the difference in chopstick-operation skill (speed and smoothness) and Oxy-Hb concentration in each ROI between pre- and post-training. We then performed a correlation analysis between the difference in chopstick-operation skill and Oxy-Hb concentrations in each ROI using Pearson’s product-moment correlation coefficient. A cross-object correlation analysis of completion time for sponge transferral with brain activity during marble transferral was also performed to assess whether the brain activity during marble transferral broadly reflected not only object-specific skills, but also more general chopstick-operation skills, irrespective of the task difficulty.

Furthermore, to interpret the results of association between the chopstick-operation skill and Oxy-Hb concentrations in each ROI more precisely, we additionally examined the relationship between the two chopstick-operation skills (speed and smoothness), pre- and post-training, in the training group, because the targeted joint movements can characterise the object transferral process, but not the chopstick operation. All statistical analyses were performed using SPSS 22.0 (IBM-SPSS Inc., Chicago, IL, USA), and statistical significance was set to 0.05.

## Supplementary information

Supplementary Information.

## Data Availability

The datasets generated and/or analysed during the current study are available from the corresponding author upon reasonable request.
