# Supplementary material for: Acquisition of chopstick-operation skills with the non-dominant hand and concomitant changes in brain activity
Source: Sci Rep. 2019 Dec 31;9:20397. doi: 10.1038/s41598-019-56956-0 (PMC6938489; doi:10.1038/s41598-019-56956-0)
Supplement: Supplementary file 1 — Supplementary Information. [file 41598_2019_56956_MOESM1_ESM.pdf]

Supplementary Information for

Acquisition of chopstick operation skills with the non-dominant hand and concomitant changes in brain activity

Daisuke Sawamura[1*], Satoshi Sakuraba[2], Yumi Suzuki[3], Masako Asano[2], Susumu Yoshida[2], Toshihiro Honke[2], Megumi Kimura[2], Yoshiaki Iwase[2], Yoshitaka Horimoto[4], Kazuki Yoshida[1], Shinya.Sakai[1]

[1]Department of Functioning and Disability, Faculty of Health Sciences, Hokkaido University, Hokkaido, Japan
[2]Department of Rehabilitation Sciences, Health Sciences University of Hokkaido, Hokkaido, Japan
[3]Department of Occupational therapy, Yamagata Prefectural University of Health Sciences, Yamagata, Japan
[4]Department of Physical Therapy, Chiba prefectural university of health sciences, Chiba, Japan

***Corresponding author:** Daisuke Sawamura
Department of Functioning and Disability, Faculty of Health Sciences, Hokkaido University, Sapporo, Hokkaido, 060-0812, Japan
Tel. & FAX: +81 011 706 3387
E-mail address: D.sawamura@pop.med.hokudai.ac.jp

Supplementary Table 1. The results of two-way and one-way ANOVAs for behavioural and Oxy-Hb concentration changes.

Measurement	Training group			Control group			Two-way ANOVA						One-way ANOVA	
							Time		Group		Interaction		Time	
	Pre-assessment	Mid-point assessment	Post-assessment	Pre-assessment	Mid-point assessment	Post-assessment	F	p	F	p	F	p	F	p
Chopstick operation skill														
Completion time of item transfer task														
Sponge (s)	12.80 (0.50)	9.52 (1.92)	8.22(0.40)	12.62 (0.60)		12.57 (0.64)	28.16	< 0.01	12.89	< 0.01	25.79	< 0.01	31.21	< 0.01
Marble (s)	70.60 (10.04)	45.72 (5.83)	32.60 (3.95)	67.43 (5.61)		70.96 (7.85)	5.70	0.02	5.88	0.02	8.27	< 0.01	10.48	< 0.01
RMSJ of upper extremity														
Shoulder abduction-adduction (degree/s^3)	22.42 (1.55)	16.02 (1.55)	14.24 (0.55)	19.51 (1.03)		16.93 (1.23)	21.60	< 0.01	0.10	0.92	5.88	0.02	19.27	< 0.01
Shoulder flexion-extension (degree/s^3)	16.43 (0.79)	14.17 (0.73)	11.77 (0.33)	14.94 (0.80)		15.38 (0.79)	9.05	< 0.01	2.22	0.15	13.14	< 0.01	18.61	< 0.01
Elbow flexion-extension (degree/s^3)	24.79 (1.60)	18.75 (0.86)	15.56 (0.94)	22.90 (1.60)		21.11 (1.21)	22.92	< 0.01	1.54	0.22	10.47	< 0.01	21.00	< 0.01
Oxy-Hb changes of ROIs														
Left DLPFC [m(mol/l*mm)]	0.024 (0.004)	0.007 (0.004)	0.005 (0.003)	0.022 (0.005)		0.022 (0.005)	4.73	0.04	1.53	0.23	5.12	0.03	8.24	< 0.01
Right DLPFC [m(mol/l*mm)]	0.022 (0.005)	0.015 (0.005)	0.012 (0.004)	0.020 (0.011)		0.21 (0.007)	0.38	0.54	0.28	0.60	0.42	0.52	1.31	0.29
Left dPMC [m(mol/l*mm)]	0.011 (0.010)	0.035 (0.006)	0.040 (0.006)	0.015 (0.009)		0.013 (0.008)	3.37	0.08	1.73	0.20	4.26	0.048	8.94	< 0.01
Right dPMC [m(mol/l*mm)]	0.018 (0.008)	0.027 (0.013)	0.043 (0.006)	0.018 (0.008)		0.017 (0.006)	4.58	0.04	2.23	0.15	4.43	0.04	5.78	0.02
Left SM [m(mol/l*mm)]	0.023 (0.005)	0.013 (0.006)	0.014 (0.004)	0.022 (0.008)		0.025 (0.008)	0.20	0.66	0.64	0.43	0.74	0.40	1.90	0.17
Right SM [m(mol/l*mm)]	0.020 (0.005)	0.009 (0.008)	0.011 (0.004)	0.019 (0.007)		0.016 (0.008)	1.19	0.29	0.09	0.77	0.28	0.60	1.80	0.19

A one-way analysis of variance with time as a factor (pre-assessment vs. mid-point-assessment vs. post-assessment) was only applied to the training group. DLPFC: dorsolateral prefrontal cortex; dPMC: dorsal pre-motor cortex; SM: primary sensory motor cortex. The values in parenthesis show the standard error.

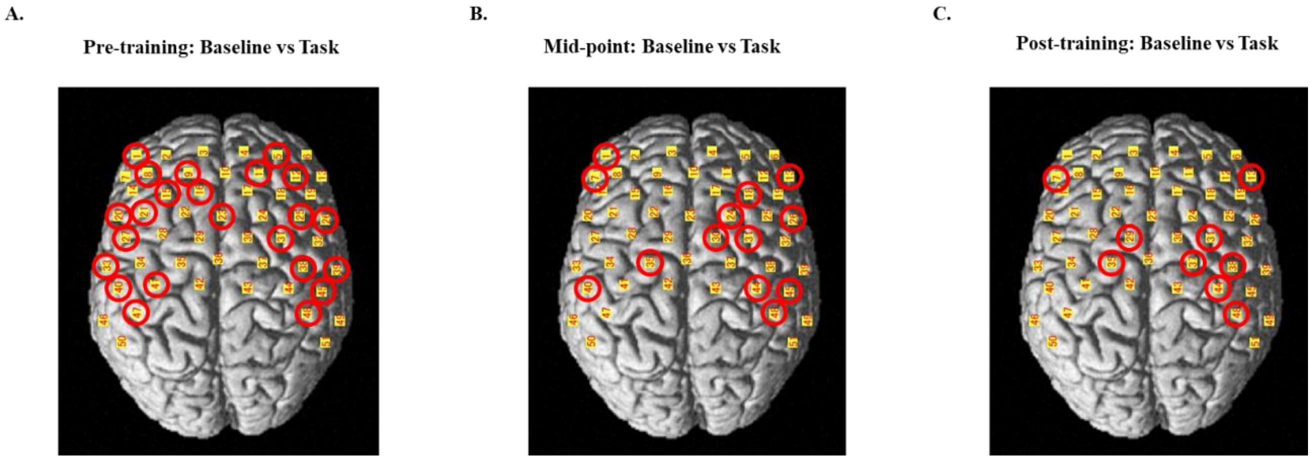

Supplementary Figure 1. Channels activated during the chopstick task. Circles indicate the activated channels during the chopstick task relative to baseline activity ($p < 0.05$, FDR-corrected) at pre-assessment (left), mid-point (middle), and post-assessment (right).

Supplementary Table 2. The correlation between the two chopstick-operation skills (speed and smoothness) at pre- and post-assessment in the training group

Measurement	RMSJ of upper extremity					
	Shoulder abduction-adduction		Shoulder flexion-extension		Elbow flexion-extension	
	Pre-assessment	Post-assessment	Pre-assessment	Post-assessment	Pre-assessment	Post-assessment
Completion time of item transfer task						
Sponge	0.54 *	0.74 **	0.07	0.54 *	0.21	0.45
Marble	-0.28	0.54 *	-0.20	0.37	0.13	0.55 *

Pearson's product moment correlation coefficient was only applied to the training group. *p < 0.05; **p < 0.01.

RMSJ: root mean square jerk

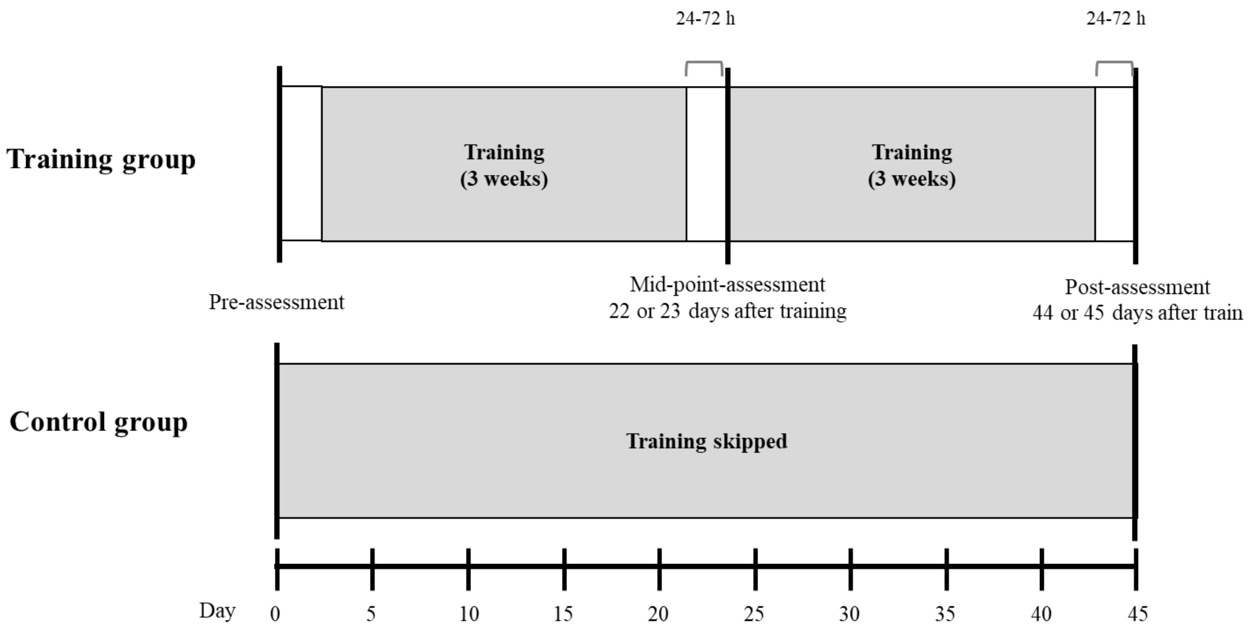

Supplementary Figure 2.

Experimental protocol in both groups. Bold vertical bars indicate the evaluation timing. Evaluations of chopstick-operation skill and brain activity were conducted three times (pre-, mid-point, and post-assessment) in the training group and two times (pre- and post-assessment) in control group. The mid-point and post-training assessment were conducted at 24 to 72 h after training to evaluate the retention of chopstick operation skills.

Supplementary Table 3. Anatomical labelling of NIRS channel positions

NIRS channel	Brodmann area	Description
1, 6	10, 46	Frontopolar area, Dorsolateral prefrontal cortex
2, 5	8, 9	Frontal eye fields, Dorsolateral prefrontal cortex
3, 4	8, 9	Frontal eye fields, Dorsolateral prefrontal cortex
7, 13	45, 46	Pars triangularis Broca's area, Dorsolateral prefrontal cortex
8, 12	8, 9	Frontal eye fields, Dorsolateral prefrontal cortex
9, 11	8	Frontal eye fields
14, 19	9, 46	Dorsolateral prefrontal cortex
15, 18	8	Frontal eye fields
16, 17	6, 8	Pre-Motor and Supplementary Motor Cortex, Frontal eye fields
20, 26	9, 46	Dorsolateral prefrontal cortex
21, 25	6, 8	Pre-Motor and Supplementary Motor Cortex, Frontal eye fields
22, 24	6, 8	Pre-Motor and Supplementary Motor Cortex, Frontal eye fields
27, 32	6, 8	Pre-Motor and Supplementary Motor Cortex, Frontal eye fields
28, 31	6	Pre-Motor and Supplementary Motor Cortex
29, 30	6	Pre-Motor and Supplementary Motor Cortex
33, 39	4, 6	Primary Motor Cortex, Pre-Motor and Supplementary Motor Cortex
34, 38	4, 6	Primary Motor Cortex, Pre-Motor and Supplementary Motor Cortex
40, 45	1, 2, 3	Primary Somatosensory Cortex
41, 44	3, 4	Primary Motor Cortex, Primary Somatosensory Cortex
42, 43	3, 4	Primary Motor Cortex, Primary Somatosensory Cortex
46, 49	40	Primary Motor Cortex, Primary Somatosensory Cortex
47, 48	1, 2, 3	Primary Somatosensory Cortex
50, 51	40	Supramarginal gyrus part of Wernicke's area
